# Purification and characterization of recombinant human bile salt-stimulated lipase expressed in milk of transgenic cloned cows

**DOI:** 10.1371/journal.pone.0176864

**Published:** 2017-05-05

**Authors:** Yuhang Wang, Fangrong Ding, Tao Wang, Wenjie Liu, Susanne Lindquist, Olle Hernell, Jianwu Wang, Jing Li, Ling Li, Yaofeng Zhao, Yunping Dai, Ning Li

**Affiliations:** 1 State Key Laboratory for Agrobiotechnology, College of Biological Sciences, China Agricultural University, Beijing, P. R. China; 2 Department of Clinical Sciences, Pediatrics, Umeå University, Umeå, Sweden; Instituto Butantan, BRAZIL

## Abstract

Bile salt-stimulated lipase (BSSL) is a lipolytic digestive enzyme with broad substrate specificity secreted from exocrine pancreas into the intestinal lumen in all species and from the lactating mammary gland into the milk of some species, notably humans but not cows. BSSL in breast milk facilitates digestion and absorption of milk fat and promotes growth of small for gestational age preterm infants. Thus, purified recombinant human BSSL (rhBSSL) can be used for treatment of patients with fat malabsorption and expressing rhBSSL in the milk of transgenic cloned cows would therefore be a mean to meet a medical need. In the present study, a vector pBAC-hLF-hBSSL was constructed, which efficiently expressed active rhBSSL in milk of transgenic cloned cows to a concentration of 9.8 mg/ml. The rhBSSL purified from cow milk had the same enzymatic activity, N-terminal amino acid sequence, amino acid composition and isoelectric point and similar physicochemical characteristics as human native BSSL. Our study supports the use of transgenic cattle for the cost-competitive, large-scale production of therapeutic rhBSSL.

## Introduction

Bile salt-stimulated lipase (BSSL)[1], which is also known as carboxyl ester lipase (CEL), bile salt-dependent lipase (BSDL) or carboxyl ester hydrolase (CEH), is secreted from the exocrine pancreas into the intestinal lumen in all species examined to date[2, 3]. It is also secreted by the mammary gland in some species, such as humans[4], but not cows or goats[5].

BSSL has broad substrate specificity with the capacity to hydrolyze tri-, di-, and mono-glycerides; cholesteryl- and retinyl-esters; fat-soluble vitamin esters; phospholipids; and ceramides[6–9]. Human milk BSSL was first described as a lipase, that when activated by primary bile salts in the duodenum contributes to efficient digestion of the milk fat. Studies in vitro[10] and in rodents[11] showed that BSSL and pancreatic lipase-related protein (PLRP)-2 are the two key lipases in intestinal fat digestion. In newborn infants, in particular preterm infants, exocrine pancreas is not fully developed[12], which suggested milk BSSL to be an important source of BSSL explaining the efficient fat digestion and absorption from human milk. BSSL is completely inactivated by pasteurization[13], which is regularly used in human milk banks and when preterm infants are fed donor milk. When preterm infants were fed pasteurized human milk fat absorption as well as weight gain are reduced as compared to when fed raw human milk[14, 15]. BSSL is also absent from infant formulas. Therefore, recombinant human BSSL (rhBSSL) was developed as an oral therapeutic strategy to improve lipid absorption and growth in non-breastfed preterm infants. Animal models had shown that rhBSSL expression in the milk of mice improved survival and growth status of preterm mice[16], and that purified human milk BSSL supplemented in formula, compared with formula alone, doubled the growth rate of kittens [17] and also that BSSL deficiency in milk caused incomplete digestion of the milk triglycerides resulting in lipid accumulation and consequent intestinal damage in neonatal mice[18]. Therefore it was not surprising that a phase II clinical trial in preterm infants showed promising results, i.e., that adding rhBSSL to infant formula or pasteurized breast milk significantly improved growth velocity and absorption of long-chain polyunsaturated fatty acid (LCPUFA) absorption [19]. However, surprisingly a recent large multicenter phase III clinical trial in preterm infants confirmed the positive effect on weight gain only in a subgroup of small for gestational age (SGA) infants[20] but not the entire study group of preterm infants. This however does not exclude a role of rhBSSL for treatment of fat malabsorption for pathological reasons. In fact, BSSL should be ideally suited for replacement therapy in conditions of exocrine pancreatic insufficiency. It is designed for per oral administration; hence, it is stable at pH above 3 and only slowly inactivated by pepsin[21]. Thus, given with a meal it will pass through the stomach virtually without inactivation and when mixed with bile in the duodenum it is not only activated by primary bile salts but bile salts also protect it from inactivation by the endogenous proteases and, as mentioned it has a broad substrate specificity[22].

In addition to its role in fat digestion, BSSL may have antimicrobial effects. Human milk BSSL, which contains α1-2-linked fucose, can act as a decoy receptor and prevent norovirus attachment to gastroduodenal tissue[23]. Furthermore, the Lewis X epitope on BSSL binds to dendritic cell-specific intercellular adhesion molecule-3-grabbing non-integrin (DC-SIGN) and prevents the transfer of human immunodeficiency virus (HIV)-1 to cluster of differentiation (CD)4+ T cells[24, 25]. BSSL also inhibits binding of the oral pathogen *Streptococcus mutans* to saliva and salivary agglutinin-coated (gp340 glycoprotein) hydroxyapatite[26]. Finally, BSSL is also found in the blood[27], but the origin and role of BSSL in the circulation is not yet fully understood but it emphasizes that BSSL indeed may have more than one function. In summary, BSSL plays a positive protective role in the intestine by promoting efficient digestion of milk fat and most likely by antimicrobial effects.

Here, we provide the first report on producing rhBSSL by transgenic cloned cows. The expression level reached 9.8 mg/ml. Cows are believed to be ideal bioreactors for high-quality and high-quantity production of recombinant proteins. When comparing rhBSSL purified from transgenic milk with native BSSL purified from human milk and rhBSSL expressed by CHO cells we found no notable differences in physicochemical and other characteristics.

## Materials and methods

### Ethics statement

The experimental cows were housed in a free stall with free access to drinking water. All the animal work in this study including the establishment of primary fetal fibroblasts were approved by the animal ethics committee of State Key Laboratory of Agro-biotechnology (license number SKLAB-2016-05-02). All experimental procedures were performed in accordance with the guidelines for the Care and Use of Laboratory Animals of China Agricultural University. During the dry period, the cows were fed whole-plant corn silage ab libitum. And during the lactation period, the cows were daily fed 8.0 kg whole-plant corn silage, and 6.0 kg commercial concentrate consisting of 530 g/kg corn meal, 140 g/kg soybean meal, 70 g/kg cottonseed meal, 40 g/kg rapeseed meal, 120 g/kg distillers dried grains, 10 g/kg limestone, 10 g/kg CaHPO4, 10 g/kg NaCl, 10 g/kg sodium bicarbonate and 10 g/kg trace mineral and vitamin premix. All efforts were made to minimise suffering of the animals.

### Construction of the hBSSL expression vector pBAC-hLF-hBSSL

Human lactoferrin BAC (hLF BAC) was used as the regulatory element. The human BSSL BAC library and hLF BAC were prepared, and *Neo* was used as a selection marker gene. First, the *Neo* gene, which is flanked by loxP sites, was electroporated into competent SW102 cells containing hBSSL BAC for recombination. After transformation, 1 ml of lysogeny broth (LB) medium was added to each tube, and the tubes incubated for 1.5 h at 30°C shaking at 220 rpm. The cells were applied to LB plates containing 50-μg/ml kanamycin. After incubation at 30°C for 24 hours, single colonies were selected and identified by polymerase chain reaction (PCR). The SW102-positive colony included BSSL-Neo BAC. Secondly, the pBR322 vector included homologous arms against BSSL-Neo-BAC, and HLF BAC was constructed with a pre-designed restriction enzyme cutting site *Not* I. Then, it was electroporated into a competent SW102 stain containing BSSL-Neo BAC for recombination. The SW102 strain was applied to LB plates with 50-μg/ml ampicillin. Using PCR, we selected the SW102 strain including PBR322-BSSL-Neo. The recombinant pBR322 plasmid was extracted and verified by *Asc* I enzyme cutting. Finally, after cutting by *Asc* I, the linear fragment containing BSSL-Neo was electroporated into the SW105 strain. Then, it was applied to LB plates with 50-μg/ml kanamycin. Single colonies were selected and identified by PCR. Positive colonies were enriched, and the recombinant plasmid pBAC-hLF-hBSSL was isolated.

### Cell culture and nuclear transfer

The cell culture and nuclear transfer procedures followed previously reported methods[28].

The plasmid pBAC-hLF-hBSSL was linearized with the restriction endonuclease *Not* I then purified by NucleoBond bacterial artificial chromosome (BAC) 100 Kit (Macherey-Nagel GmbH & Co. KG, Düren, Germany). The linearized plasmid DNA fragment was transfected into embryonic fibroblasts cells, which were isolated from a 46-day-old Holstein cow fetus, using program T-016 and Amaxa Nucleofector reagent (Lonza Group AG Basel, Switzerland). 48 hours after transfection, cells were dispersed by limiting the dilution to concentration of 300 cells per 10 cm^2^ dish. Each cell clones were then seeded into a 48-well cell culture plate for another 48 h. Then they were transferred into a 12-well cell culture plate, and selected part of them for PCR analysis. According to the Electro Cell Manipulation System (BTX, San Diego, CA, USA), the nuclear of positive cell colonies were transferred into enucleated oocytes to produce reconstructed embryos. Future transplantation was performed with Day 7 blastocysts. Embryo cryopreservation was performed with vitrification method. After freezing/thawing, 35 blastocysts were then transferred into 17 recipient Chinese Luxi yellow cows. Each recipient was transferred with one or two transgenic cloned blastocysts. Three months and eight months after transfer, ultrasonography was performed for pregnancy detection. Experiments were performed following relevant guidelines and regulations.

### PCR analysis

PCR was performed to confirm whether the rhBSSL gene was transferred to the transgenic cloned cows. The primer was BSSL P4. And primers BSSL P5-BSSL P10 were used to determine the integrity of the gene. Primer Sequences were list at S1 Table.

### Southern blot analysis

The plasmid pBAC-hLF-hBSSL and the DNA samples extracted from ear samples from transgenic and wild-type (WT) cows were digested overnight with the restriction enzyme *Nco* I (R0193S, NEB). The digoxigenin-labeled probe was amplified using the primer pair BSSL P3, and the probe length was 725 bp. The primers are as follows: F: 5’-ATGTGTTGCTTCTGGTTCCTTTCCTCC-3’ and R: 5’-CAGCTTTGACTCCGATCCTCAGTTTCC-3’. After agarose gel electrophoresis for 4 h, the DNA was transferred to a nitrocellulose filter for blotting. The nitrocellulose membrane was hybridized with a probe for 18 h and incubated with antibody for 0.5 h. The size of the positive bands was expected to be approximately 3 kb. The reagents used for the Southern blot analysis were purchased from Roche Diagnostics GmbH (Mannheim, Germany).

### Quantitative PCR (Q-PCR)

The transgene copy number was identified by Q-PCR. The primer pair BSSL copy5F/R, F: 5’-GCAAACATTTACTGAACCGTAGCA-3’ and R: 5’-AGCA AGCTGGTAAGGCTGACA-3’ amplifies a 120-bp product. Bovine *myostatin* was chosen as the internal control gene. The primer pair bovine myostatin F: 5’-TCCGTCCTGGCGTGGTAG-3’ and bovine myostatin R: 5’-GCTATCAGACAACTTTTGCCCAAG-3’ amplifies a 122-bp product. The Q-PCR reaction system and conditions were as previously reported[28]. All PCRs were performed using a Roche LightCycler 480 System (LC 480; Roche Diagnostics, Basel, Switzerland).

### Collection of transgenic milk

Milk samples were collected by induced lactation from eight-months-old transgenic cows. They were injected with medroxyprogesterone acetate (25mg/kg/day) and estradiol benzoate (7.5 mg/kg/day) for seven days. Milk sample were collected for one week from day one of lactation.

### Sodium dodecyl sulfate polyacrylamide gel electrophoresis (SDS-PAGE) and western blot analysis

For SDS-PAGE, the milk protein samples were separated on 12% Tris-glycine polyacrylamide gels under denaturing and reducing conditions, and the protein content was quantified with bicinchoninic acid (BCA) assay. For the western blot analysis, the diluted milk samples were separated on 10% Tris-glycine polyacrylamide gels under denaturing and reducing conditions and then transferred to polyvinylidene fluoride (PVDF) membranes (Invitrogen Corporation, Carlsbad, CA, USA), which were incubated with a polyclonal anti-human hBSSL antibody (dilution, 1:1,000; Catalog Number sc-34878) and a horseradish peroxidase-conjugated secondary anti-goat immunoglobulin (Ig)G antibody (dilution, 1:20,000; Sino-American Co., Beijing, China).

### Analysis of the rhBSSL expression levels

The rhBSSL expression levels in cow’s milk were measured using in house human BSSL enzyme-linked immunosorbent assay (ELISA). A monoclonal antibody against human native BSSL was placed in a coating plate overnight at 4°C with a concentration of 8 μg/ml. After the plate was washed with phosphate-buffered saline with Tween 20 (PBST; PBS, pH 7.4, 0.05% Tween 20), the plate was blocked with blocking buffer (1% bovine serum albumin[BSA] in PBST) for 2 hours. The standard samples were a native human BSSL concentration series of 4000 to 0 pg/ml. The samples were diluted at the appropriate dilution factors and incubated for 2 hours. After washing, another conjugate polyclonal antibody against human BSSL was added at a concentration of 1 μg/ml and incubated for 1 hour. The plate was washed 4 times, and then, 100 μl of tetramethylbenzidine (TMB) substrate was added to each well and incubated for 15 min. The reaction was stopped with 50 μl of 1-M H_2_SO_4_ in each well. Then, the OD450 value was read with a micro-plate reader. All of the procedures were performed at room temperature.

### Determination of BSSL activity

The activity was performed as follows. 40μl labeled ^3^H-triacylglycerol (PerkinElmer, NET431001MC) was mixed with 25 mg of unlabeled triacylglycerol (Sigma, #T7140) and evaporated under N_2_ for 10 min; then, added with 1.0 ml of 10% gum arabic, 1.25 ml of 1.0-M Tris/HCl buffer (pH 9.0), and 2.0 ml of distilled water. The mixture was cooled with ice water and sonicated for 5 min for maximal effect in a 100-W disintegrator (MSE ltd, London, England). To this emulsion, 2.5 ml of 1.0-M NaCl, 2.5 ml of 18.7% defatted BSA and 3.25 ml of deionized water were added. The assay tubes containing 150 μl of this medium and 10 μl of 200 mM Sodium Cholate (sigma) and enzyme source in a total volume of 200 μl were incubated at 37°C in a water bath shaking at 50 strokes per min. The reaction was stopped after 15 min by the addition of 3.25 ml of a methanol: chloroform: heptane mixture (1.41:1.25:1 [V/V/V]), immediately followed by 1 ml of 0.1-M potassium carbonate buffer, pH 10.5. The tubes were vigorously shaken and centrifuged at 2250 rpm for 10 min in a Sorval GLC-1 centrifuge (HL-4 rotor). The fatty acids were extracted to the upper phase of which 0.4 ml was sampled in a counting vial containing 2 ml of aquasol. The radioactivity was determined in a liquid scintillation spectrometer (Packard Tri-Carb model 3020).

### Purification of rhBSSL

Purified by Heparin-Sepharose chromatography was performed based on previously work[29]. Modifications were made as follows. Milk was centrifuged at 2500 rpm for 20 min at 4°C to remove the fat. The skimmed milk was adjusted to pH 4.6 to precipitate casein and centrifuged at 100,000 × g at 20°C for 1 h. Purification initially involved Heparin Sepharose Chromatography (HiTrap Heparin HP). First, the column was equilibrated with equilibration buffer (10-mM phosphate buffer, pH 7.0); then, the samples were loaded into a HiTrap Heparin HP (GE Healthcare, 5 ml), and the protein was eluted with a linear gradient of 0–1-M NaCl in 10-mM phosphate buffer, pH 7.0. To improve the purity of the rhBSSL, a gel-filtration matrix (Superdex 200) was used with 10-mM phosphate buffer, pH 7.0. The purified rhBSSL was further detected by SDS-PAGE, western blotting and ELISA.

### Characterization of rhBSSL

#### Molecular weight, isoelectric point and N-terminal amino acid sequence

The purified rhBSSL and human native BSSL were sent to Shanghai Applied Protein Technology Co., Ltd (Shanghai, China) for molecular weight assay by matrix-assisted laser desorption/ionization time of flight mass spectrometry (MALDI-TOF-MS) (Bruker Daltonics, Billerica, MA, USA). Isoelectric point was analyzed by isoelectric focusing method. The N-terminal amino acid sequence of the purified rhBSSL was analyzed by automatic Edman degradation. The resulting N-terminal sequence of the test was aligned to the GenBank databases of the National Center for Biotechnology Information (GenBank accession no. AAH42510.1).

#### Bile salt-dependency, resistance to pH, heat and trypsin

Human native BSSL and CHO-produced BSSL were gift by Prof. Olle Hernell, Umeå University. Native BSSL requires primary bile salt (sodium cholate) for its activity against triacylglycerol. The BSSL activity was measured at different sodium cholate concentrations in the range of 0–14 mM. For the pH stability test, 5-μg/ml BSSL was incubated at different pH (range 2–12) buffers in the presence of 1-mg/ml BSA for 30 min. Then, BSSL activity was assessed as described. The activities are presented as the percentage of the activity for each sample at a pH of 7.4. For the heat stability test, before the activity assay, the BSSL was treated at different temperatures (37°C, 40°C, 45°C, 50°C and 55°C) for 30 min. The activities are presented as the percentage of the activity for each sample at 37°C and 0 min. For the trypsin stability test, the purified samples (1.5 μg) were added to 600 μl of 1.0-M Tris/HCl, pH 7.4, containing 100 μg of trypsin (bovine, Sigma) at 25°C in absence or presence of 10-mM sodium cholate. After incubation for 0, 10, 20, 30, and 60 min, the BSSL activity was analyzed. All values are presented as the percentage of the value obtained in the control incubation in the absence of trypsin. All activity is shown with TG as the substrate.

#### Kcat and Km determination

The Kcat and Km analysis was performed as described [30]. Para-nitrophenyl-caproate (pnpC10, Sigma, CAS 1956-10-1) was used as substrate. The experiments were repeated at least six times. Kcat and Km were determined by Graph Pad Progrom with the Michaelis-Menten model.

## Results

### Construction of the *hBSSL* expression vector pBAC-hLF-hBSSL

**Fig 1:**
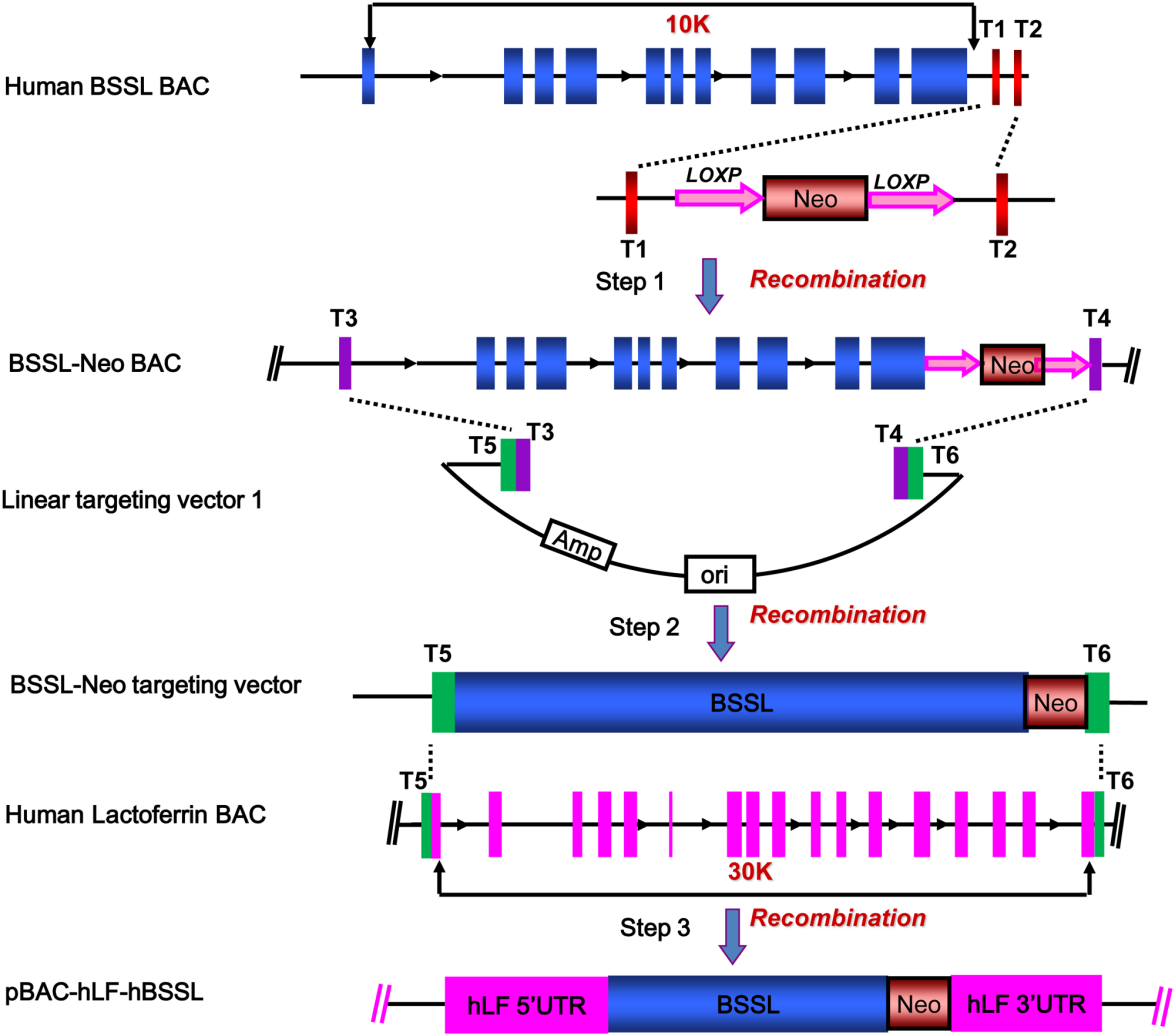
Construction of the *hBSSL* expression vector pBAC-hLF-hBSSL and strategy for replacing 28.9 kb of hLF genomic sequence with 9,8 kb of hBSSL genomic DNA on the hLF BAC. hLF BAC was used as regulating element for expression of human BSSL. By homologous recombination, the hLF genomic DNA in the hLF BAC was replaced with the 9.8-kb *hBSSL* gene. A *Neo* selection marker was downstream of BSSL. hLF BACs have a 90-kb 5’UTR and a 30-kb 3’UTR, and they were used as transcriptional regulating elements. The procedure of modifying the pBAC-hLF-hBSSL construct was performed by three-step capture method. Each step was verified by PCR and sequencing with the primers. The first step, A pair of homology arms T1 T2 were designed against downstream of BCA *BSSL* gene, then link them to *Neo* gene with loxP site at each flank. By homologous recombination we got BSSL-Neo BAC, the *Neo* resistant gene was insert into the downstream of *BSSL* and be used as prokaryotic and eukaryotic selectable marker. In the second step, homology arms T3 T4 against BSSL-Neo BAC and T5 T6 against hLF BAC were designed and then connection of those two pairs of homology arms by pre-designed restriction enzyme cutting site before link to pBR322 vector. Cutting T3 T4 by *Not* I, then we get the linear targeting vector 1. Electroporated it into competent SW102 cells containing BSSL-Neo BAC for recombination. By this step BSSL-Neo was captured and circular plasmid pBR322 containing BSSL-Neo was success prepared and we named it BSSL-Neo targeting vector. In the final step, cutting the isolated recombinant plasmid pBR322 by *Asc* I which was pre-designed at the two flank of T5 T6, collect the fraction contain BSSL-Neo and homology arms T5 T6 by gel extraction. The fragment was then electroporated into competent SW105 stains containing hLF BAC for recombination by homology arms T5 T6. Then the chimeric BAC pBAC-hLF-hBSSL was prepared. It contained a 90 kb 5’flanking region of the *hLF* gene, the 9,8-kb hBSSL genomic fragment and a 30-kb 3’ flanking region of the *hLF* gene, and a *Neo* cassette for future selection.

Here, we describe hLF BAC as a regulating element for the regulation of the expression of human BSSL (Fig 1). By a three-step homologous recombination, the hLF genomic DNA in the hLF BAC was replaced with the 9.8-kb hBSSL gene. A *Neo* selection marker was downstream of BSSL. hLF BACs have a 90-kb 5’UTR and a 30-kb 3’UTR, and they were used as transcriptional regulating elements.

### BSSL transgenic cell colony screening, transgenic cows identification and integration analysis

**Fig 2:**
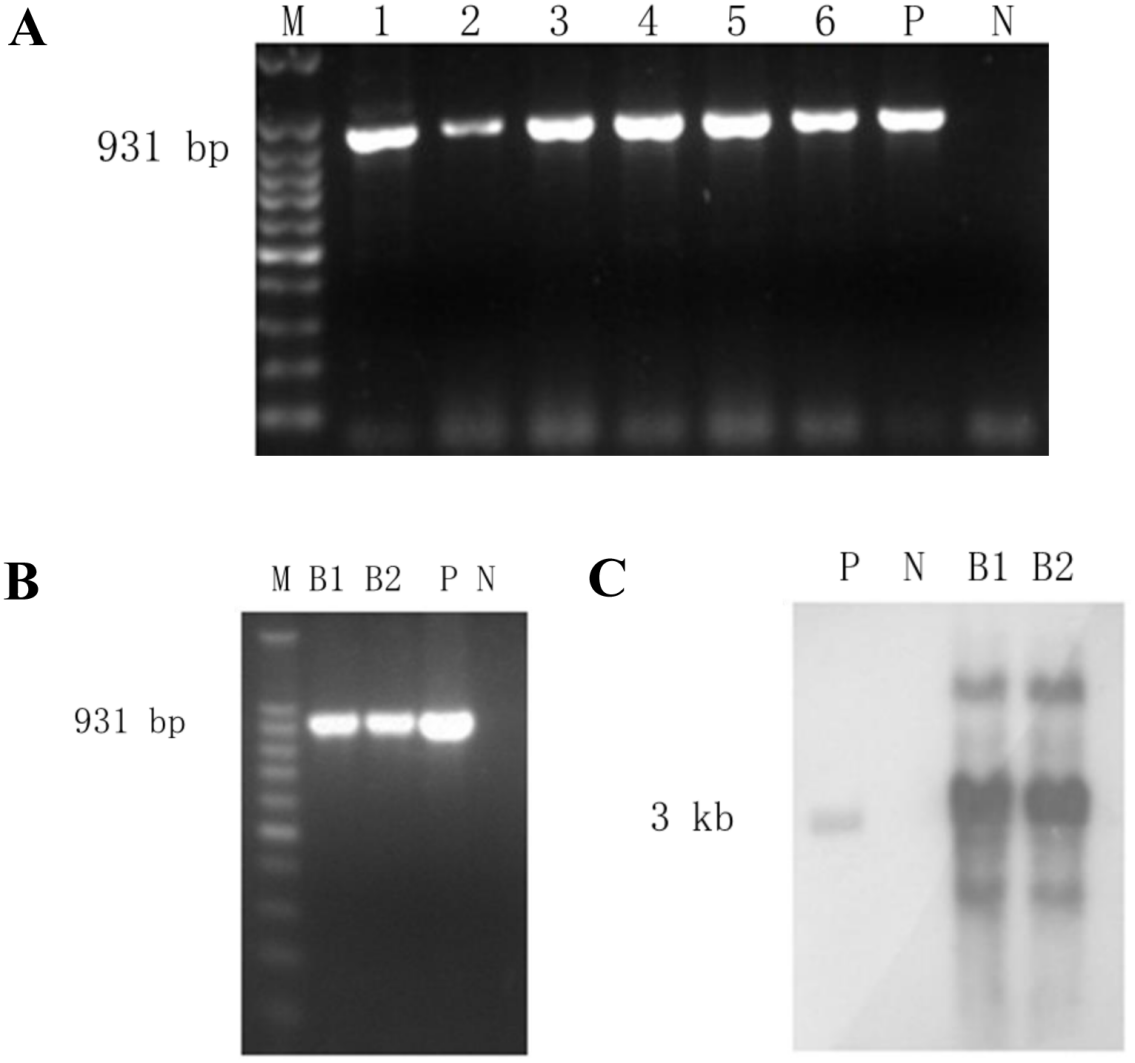
Identification of transgene in isolated cell colonies and transgenic cows. (A) 6 cell colonies were analyzed by PCR. M, 100bp DNA ladder; P, postive control construct pBAC-hLF-hBSSL; N, genomic DNA from non-transfection cells; 1–6 represent 6 cell colonies. (B) PCR analysis and (C) Southern blot identification of transgenic cloned cows. B1,B2, genomic DNA from transenic cows; P, postive control construct pBAC-hLF-hBSSL; N, genomic DNA from wildtype cows.

The expression plasmid pBAC-hLF-hBSSL was transfected into bovine fetal fibroblast cell 094FFB colonies (Table 1). PCR analysis confirmed the positive colonies, which were then used for nuclear transfer (Table 2). The integration of the human *BSSL* gene into the genome of the transgenic calves was confirmed by PCR. Six positive cell colonies were identified (Fig 2A), of which two were used as donor cells for the nuclear transfer. Ultimately, we obtained two newborn calves positive for expression BSSL confirmed by PCR (Fig 2B), which were further verified by Southern blot analysis (Fig 2C). The copy numbers of the transgenic cloned cows were identified by Q-PCR, with copy number 12 and 13 separately.

**Table 1:** Cell screening of pBAC-hLF-hBSSL transfection.

Cell line	Screening method	Operation times	Isolated colonies	Cell colonies selected for nuclear transfer	Freezing cell colonies	Cell colonies selected for nuclear transfer
094FFB	Low concentration G418	1	73	15	11	BSSL-1#
094FFB	Low concentration G418	2	421	21	16	BSSL-2#

Cell line 094FFB was transfected with linear expression plasmid pBAC-hLF-hBSSL.

**Table 2:** Summary of nuclear transfer results.

No. of cell clone	Oocytes	Re-construct embryos	Blastocysts	Recipients	Pregnacy at 3 months(%)	Birth rate
BSSL-1#	73	35	0	0	0	0
BSSL-2#	421	268	145	17	6(35%)	3(17.6%)

Birth rate of transgenic cows after nuclear transfer.

### Expression of rhBSSL in the milk of transgenic cloned cows

**Fig 3:**
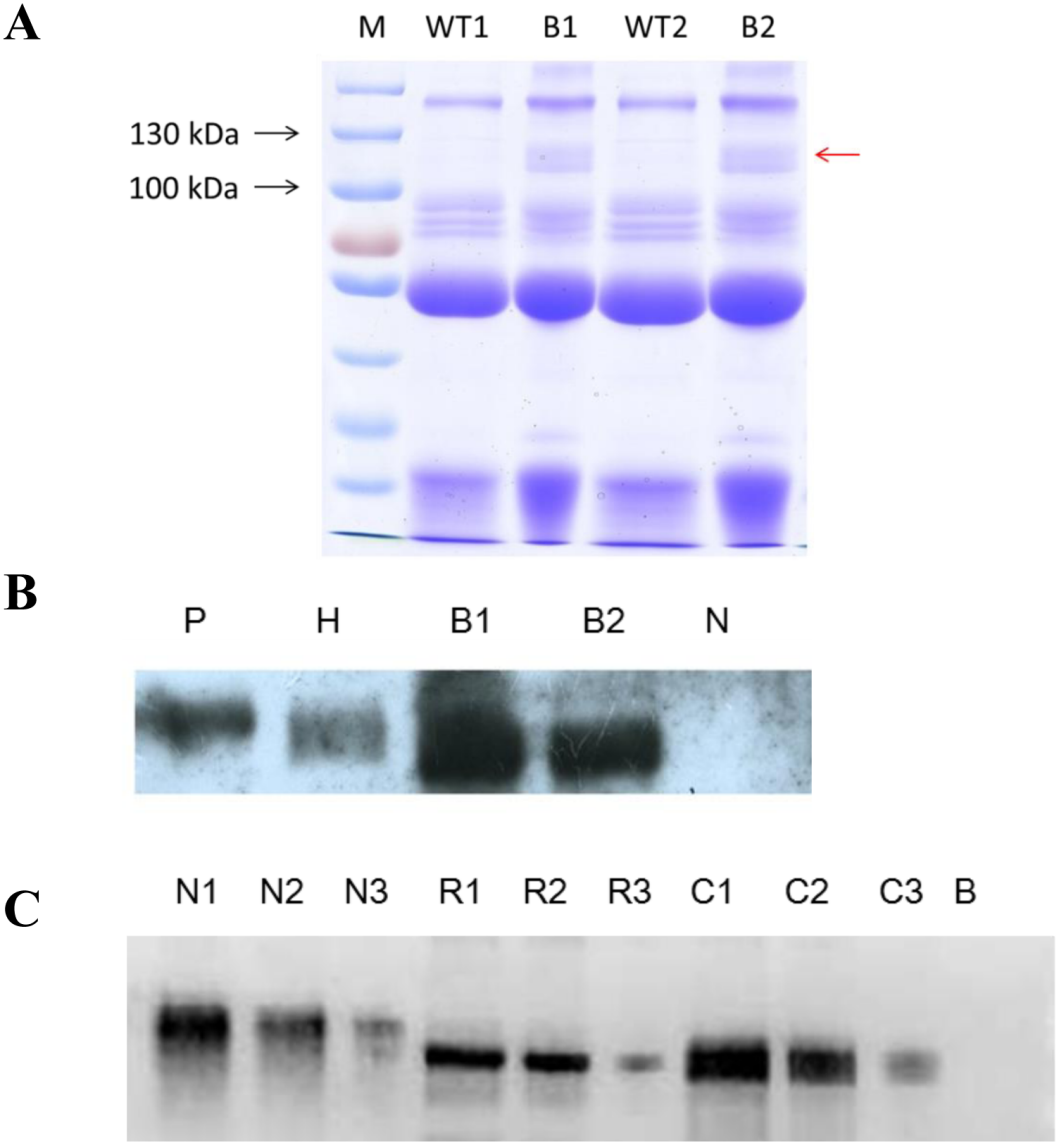
Expression of rhBSSL in transgenic milk. (A) SDS-PAGE of milk from transgenic cloned cow milk and wild type cow milk. WT1 and WT2, milk from wild type cow milk; B1 and B2, milk from transgenic cloned cows. The red row show extra band between 100–130 kDa in transgenic cow milk compare with wild type cow milk. (B) Western blot identification of rhBSSL in milk. P, purified human BSSL; H, human milk; B1, B2, milk from transgenic cloned cows. N,milk from wild type cow. (C) Comparison of purified rhBSSL from transgenic milk with native BSSL and CHO-produced BSSL by western blot. N, purified human native BSSL (nBSSL); N1, 10 ng nBSSL; N2, 5 ng nBSSL; N3, 1 ng nBSSL; R, purified rhBSSL from transgenic cow milk (rhBSSL); R1, 10 ng rhBSSL; R2, 5 ng rhBSSL; R3, 1 ng rhBSSL; C, purified rhBSSL from CHO cells (CHO-rhBSSL); C1, 10 ng CHO-rhBSSL; C2, 5 ng CHO-rhBSSL; C3, 1 ng CHO-rhBSSL; B, blank.

Milk samples from transgenic and wild type cows were collected and analyzed by SDS-PAGE and western blotting. The molecular mass of native human BSSL is approximately 100–130 kDa in size. An extra band in transgenic cow’s milk was clearly detected between 100–130 kDa, which cannot be detected in the wild type one (Fig 3A). Western blotting showed positive bands for all samples from the transgenic cloned cows, whereas none was found in wild type cow’s milk (Fig 3B). ELISA and the ^3^H -labeled radioactivity method were used to quantify the rhBSSL expression level and activity in the transgenic milk. The expression level of rhBSSL in the induced milk was 9.8 mg/ml.

### rhBSSL purification, recovery rate and purity analysis

**Fig 4:**
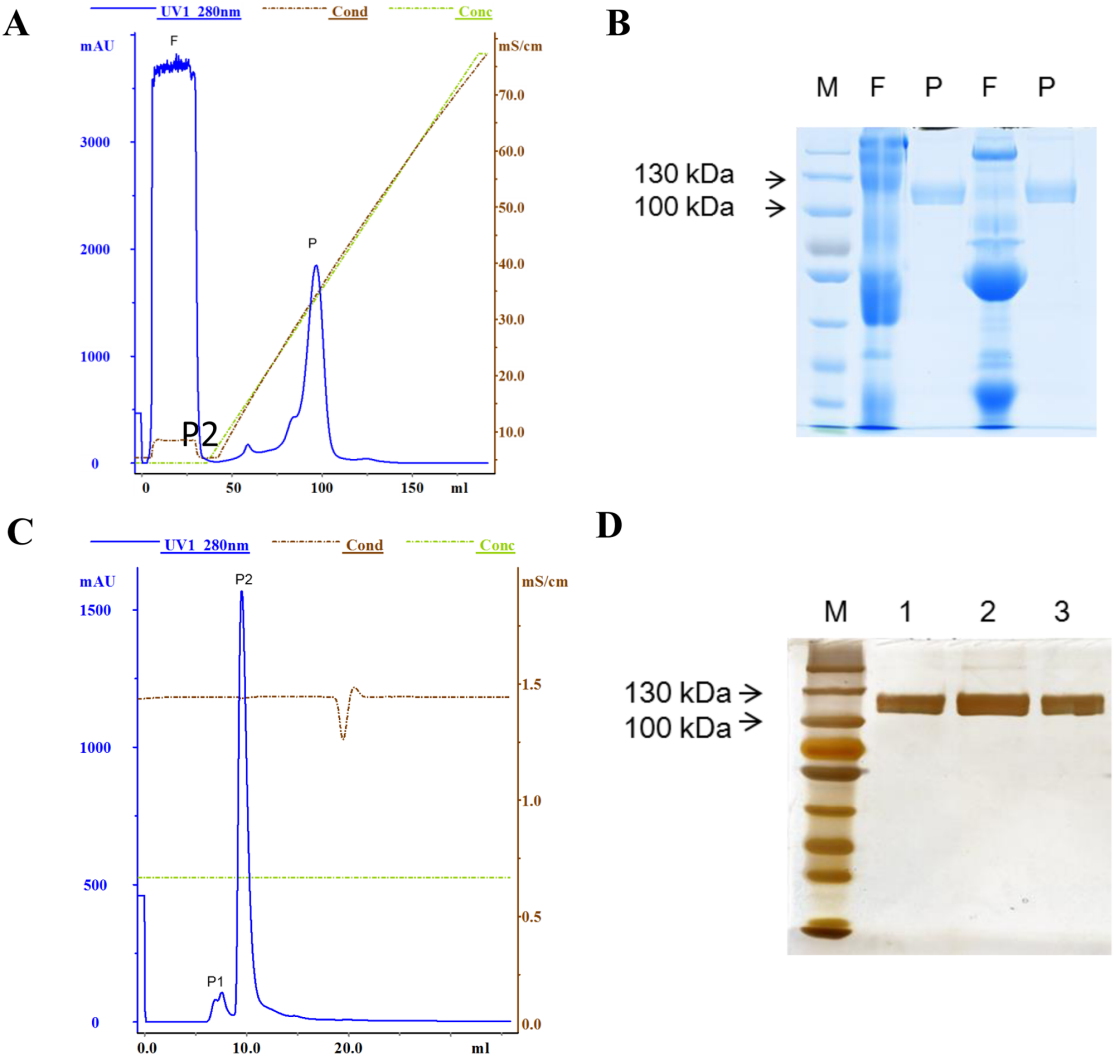
Purification and identification of rhBSSL from transgenic milk. (A) purification of rhBSSL by heparin-chromatography with HiTrap Heparin HP. 25ml milk whey was loaded onto a HiTrap Heparin HP 5ml column. F, the flow through; P, the elution peak. (B) 12% SDS-PAGE and Coomassie blue staining of the flow-through and elution peak. (C) Purification of rhBSSL from the first chromatographic step by Superdex 200 gel filtration chromatography. The P fraction from first step was loaded on the column after being desalted by 10mM sodium phosphate buffer (pH 7.0). P1, impurities and P2, purified rhBSSL. (D) Purity of rhBSSL (P2) was examined by SDS-PAGE and silver staining. 1, 6 μg rhBSSL; 2, 12 μg rhBSSL; 3, 10 μg rhBSSL.

Fat and casein of transgenic cow milk were removed by adjusting the pH to 4.0. The whey was filtered through a 0.22-μm filter and loaded onto a HiTrap Heparin HP 5-ml column. The binding buffer was 10-mM sodium phosphate, pH 7.0. Bound protein was eluted by a linear gradient of 0 to 1M NaCl in 10-mM sodium phosphate, pH 7.0. One major peak containing the BSSL protein was eluted at approximately 0.4 M NaCl (Fig 4A). The flow through and peak fraction were collected for SDS-PAGE and Coomassie blue staining (Fig 4B)**.** The purity of the collected rhBSSL was analyzed by high-performance liquid chromatography (HPLC). Super SW300 was used for the analysis. The purity was 89.7% after the first heparin chromatography. Further purification was obtained by Superdex200 gel filtration chromatography (Fig 4C), yielding 98.1% pure rhBSSL. Silver staining confirmed the purity (Fig 4D).

### Characterization of purified rhBSSL

The molecular mass of the purified rhBSSL was determined by matrix-assisted laser desorption/ionization time-of-flight mass spectrometry (MALDI-TOF-MS) to be 79800.4 Da. Thus, compared with native human BSSL (nBSSL), which had a molecular mass of 116232.4 Da, rhBSSL was smaller. However, rhBSSL from the transgenic milk have a similar size with the rhBSSL expressed by CHO cells (Fig 3C), possibly due to rhBSSL by these cells and the transgenic cows has considerably lower degree of glycosylation. The N-terminal amino acid sequence of purified rhBSSL was identical to that of the native BSSL from human milk (GenBank accession no. AAH42510.1). Furthermore, rhBSSL had the same isoelectric point as nBSSL. Enzymatic activity was analyzed with TG as substrate with no significant difference between rhBSSL and native BSSL (Table 3).

**Table 3:** Characteristics of purified rhBSSL compared with human native BSSL(nBSSL).

characteristic	nBSSL	rhBSSL
Molecular mass	116232.4 Da	79800.4 Da
N-terminal sequence	NH 2 -Ala-Lys-Leu-Gly-Ala-Val-Tyr-Thr-Glu-Gly-Gly-Phe-Val-Glu-Gly	NH 2 -Ala-Lys-Leu-Gly-Ala-Val-Tyr-Thr-Glu-Gly-Gly-Phe-Val-Glu-Gly
Isoelectric point	4,0	3.99
Enzymatic activity(mU/mg)	102.7±4.8	102.1±7.2

### Bile salt dependency and stability of rhBSSL

**Fig 5:**
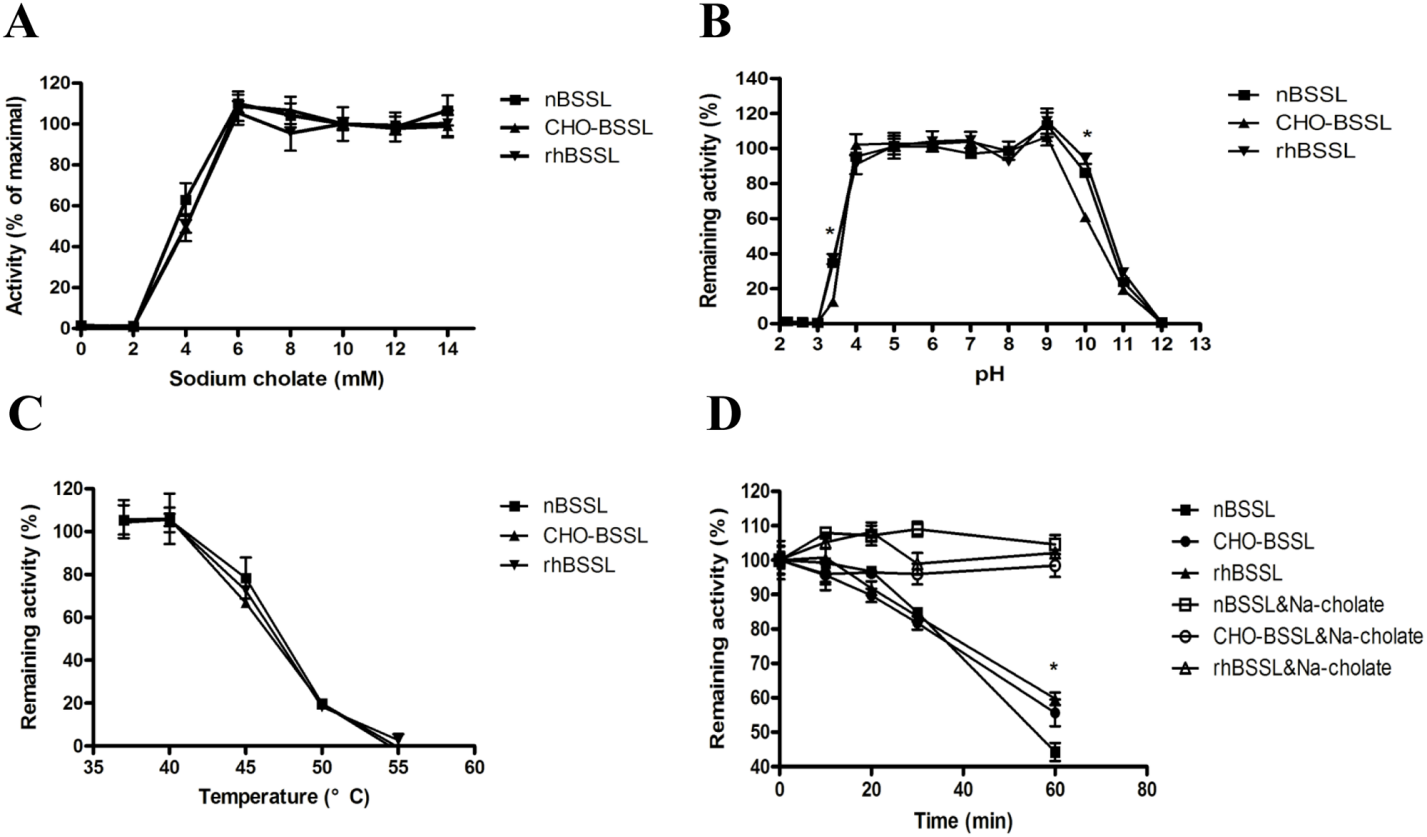
Bile salt dependency and stability of rhBSSL. A. Bile salt dependency of BSSL. BSSL activity was measured at sodium cholate concentrations 0, 2, 4, 6, 8, 10, 12 and 14 mM. Values are present as the percentage of activity under 10 mM sodium cholate. B. Stability of BSSL at different pH. BSSL were incubated at 37°C for 30 min in buffer containing 1mg/ml bovine serum albumin with pH ranging from 2 to 12. Aliquots were withdrawn and assayed for lipase activity. Values are present as the percentage of activity at pH7.0. CHO-rhBSSL is less stable than native and rhBSSL at pH3.4 and pH10. C. Heat stability of BSSL. BSSL were incubated in 50 mM Tris/HCl, pH 7.5 containing 1 mg/ml bovine serum albumin at the temperatures indicated, 37°C, 40°C, 45°C, 50°C and 55°C. After 30 min incubation, samples were withdrawn and assayed for lipase activity. Values are present as the percentage of activity at 37°C. D. Effect of bile salts on the inactivation of BSSL by trypsin. Purified samples (1.5μg) were added to 600μl 1.0M Tris/HCl, pH 7.4 with 100 μg trypsin at 25°C absence and presence of 10 mM sodium cholate. At the times indicated, aliqots were withdrawn and assayed for lipase activity. Values are expressed as the percentage of values obtained in control incubations in absence of trypsin. rhBSSL and CHO-rhBSSL are more resistance to trypsin after incubate 60 min. All the experiment were repeated at least three times, and the results were presented as mean ± SD. * means p<0.05, student’s t-test.

rhBSSL was compared with human native BSSL (nBSSL) and CHO cell-expressed BSSL (CHO-BSSL) with respect to bile salt dependency and stability towards different pH, heat and trypsin. BSSL requires primary bile salt (sodium cholate or chenodeoxycholate) for activity against long-chain TG emulsions. For bile salt dependency assessment the BSSL activity was analyzed at sodium cholate concentrations ranging from 0 to 14 mM. The activity of BSSL was optimal in the presence of a minimum of 6 mM sodium cholate (Fig 5A). To test the pH stability, rhBSSL, nBSSL and CHO-BSSL were first incubated for 30 min at different pH (pH 2–12) for 30 min where after BSSL activity assays were performed. The results showed that all three sources of BSSL were stable between pH 4 to 9. However, inactivation occurred at pH values below 4 and above 9, and CHO-BSSL seemed to be slightly more sensitive to low or high pH (Fig 5B). The heat stability test showed that they all gradually lost activity above 45°C and that incubation at 55°C for 30 min resulted in a complete loss of activity (Fig 5C). In summary, BSSL is sensitive to heat, and the pasteurization of transgenic milk or exposure to trypsin gradually destroys the activity. Human native BSSL is sensitive to trypsin as is the rhBSSL, although the latter seems more resistant to trypsin digestion than does the nBSSL. However, in the presence of bile salt both enzymes are protected from this degradation, and the activity thus maintained (Fig 5D). No notable differences in the characteristics were found between the rhBSSLs or between these and nBSSL.

### Kinetic behaviour of BSSL with pnpC10 as substrates in the presence of sodium cholate

**Fig 6:**
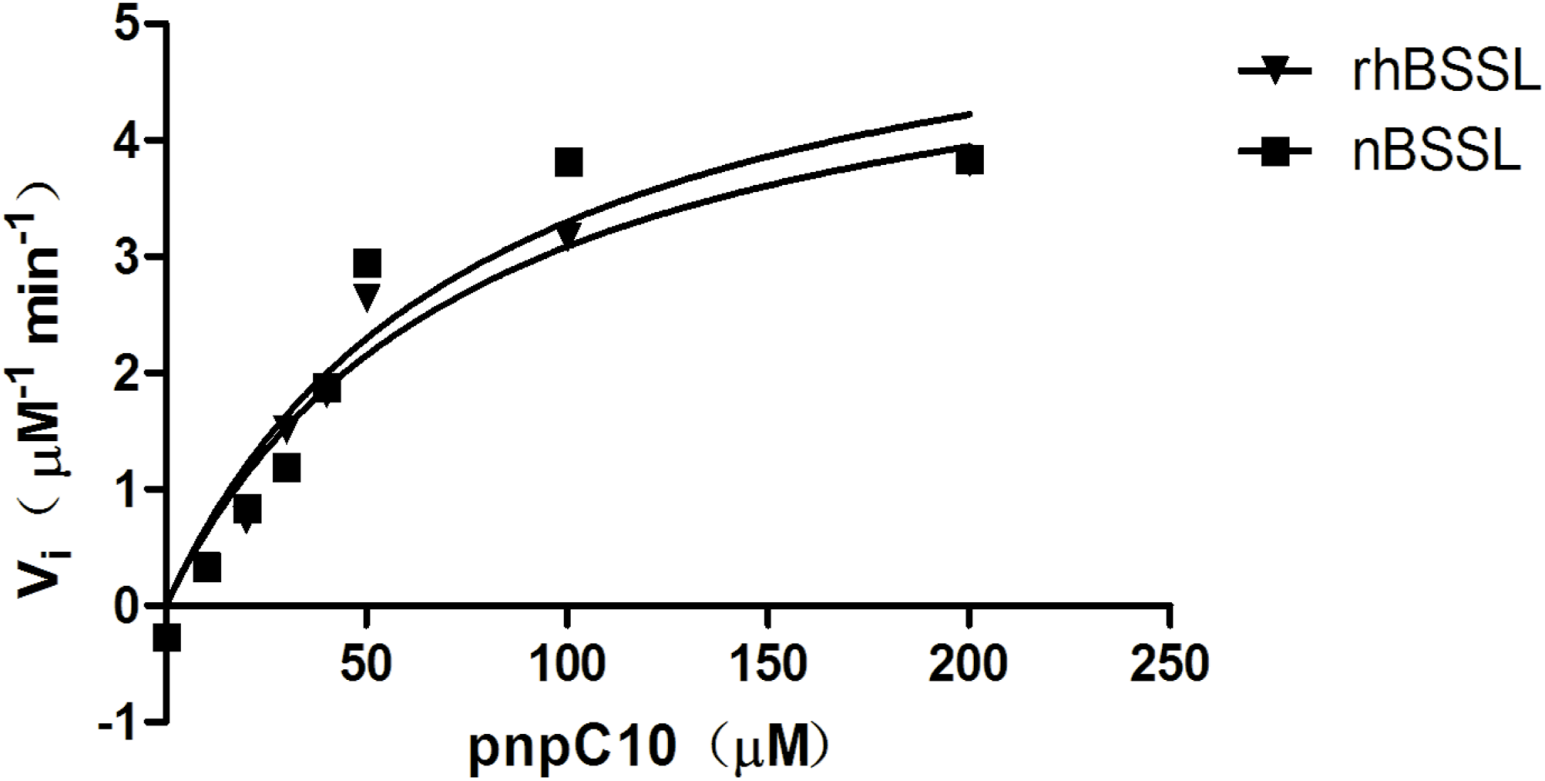
Michaelis Menten graphs for rhBSSL and nBSSL with pnpC10 as substrates, with 10 mM sodium cholate. The velocity of release of pnp was calculated as initial velocity (vi expressed as μM/min). The substrate pnpC10 concentrations were from 0 μM to 200 μM. Data are present under 10 mM sodium cholate. Km of rhBSSL and nBSSL were 77±23 μM vs 77±33 μM.

Plots of initial velocity versus pnpC10 concentration with 10 mM sodium cholate, curves of rhBSSL and nBSSL well fit the Michaelis-Menten model are shown in Fig 6. Km of rhBSSL and nBSSL were 77±23 μM vs 77±33 μM, respectively while the respective Kcat of rhBSSL and nBSSL were 55±8 s^-1^ vs 59±12 s^-1^.

## Discussion

In the present study, a vector pBAC-hLF-hBSSL was constructed, which efficiently expressed active rhBSSL in milk of transgenic cloned cows with a concentration of 10 mg/ml. The rhBSSL purified from cow milk had the same specific lipase activity, N-terminal amino acid sequence, isoelectric point and physicochemical characteristics as human native BSSL. Our study thus supports the use of transgenic cattle for large-scale production of rhBSSL for therapeutic use.

Cow milk and breast milk differ substantially in their composition. Disregarding a continuous development of infant formulas breast-fed infants still exhibit better performance in several outcomes than formula-fed infants[31]. Modern infant formulas, of which most are based on cow milk protein, were developed using the composition of human milk as a reference[32]. Bioactive substances present in human milk but not bovine milk are ingredients of interest for the production by transgenic cloned cows. BSSL is one of these substances, and it has an important effect on fat digestion and energy utilization, particularly in preterm infants[33]. In this study, we provide the first report of expression of human BSSL in transgenic cow’s milk.

Human BSSL is not abundant in breast milk relative to other multifunctional proteins in human milk, such as lactoferrin[34]. The level of BSSL in human milk is approximately 100–200 μg/ml[35]. Nevertheless, human BSSL plays an important role in milk fat absorption, together with PLRP2 and gastric lipase[36]. Over the past two decades, rhBSSL has been found to be expressed in C127 cells[37], Chinese hamster ovary (CHO) cells[21], Pichia pastoris[38, 39] and transgenic mice[16, 40, 41] and sheep[42]. For the large-scale collection of rhBSSL, transgenic sheep and CHO cells producing rhBSSL have the potential to be developed for commercial production. Transgenic sheep were generated by genomic BSSL regulated by the beta-lactoglobulin (BLG) promoter. The expression levels were in excess of 3 g/L in transgenic sheep milk[42]. The rhBSSL in transgenic sheep milk was fully active and exhibited the same enzyme characteristics as the native BSSL. Human BSSL expressed and purified from CHO cells has been developed for medicinal use to ameliorate fat malabsorption in preterm infants. Clinical phase II trials have shown that the addition of rhBSSL to formula or pasteurized breast milk significantly improves the growth rate of preterm infants[19]. Furthermore, rhBSSL improved the absorption of the long-chain polyunsaturated fatty acids docosahexaenoic acid and arachidonic acid which are important fatty acids for infant neurodevelopment[43]. However, the recent phase III study confirmed the positive effect on weight gain only in the subgroup of small for gestational age (SGA) infants[20].

The cattle mammary bioreactor has been developed for the large-scale production of functional human milk proteins[28, 44–46]. Here, we sought to produce high-efficiency and active rhBSSL in cow milk using this principal technique. Optimizing the expression construct was essential to achieve high expression of rhBSSL. In a previous study, we used a beta-casein promoter as the regulatory sequence coupled with a human BSSL cDNA sequence. rhBSSL was expressed in transgenic mice up to 377μg/ml[16]. The use of cDNA may explain the low expression level of BSSL. The genomic DNA of BSSL has been shown to raise the expression level in the mammary gland above that of BSSL cDNA. To improve the rhBSSL level in milk, a new expression vector—pBAC-hLF-hBSSL—was constructed. The hLF-BAC regulating construct has the advantage of fully integrated regulatory elements, thus facilitating efficient expression of the recombinant protein. In the induced milk of transgenic cloned cows, the rhBSSL expression level increased to approximately 10 mg/mL confirming that pBAC-hLF-hBSSL is an efficient construct to induce rhBSSL production in milk of transgenic cloned cows. When compared with the method used for obtaining rhBSSL from commercial CHO cells, the advantage of our method is that rhBSSL can be harvested several times per day at a larger scale and it can be readily prepared from cow’s milk.

Methods of purifying BSSL from human milk have been developed as previously research[29, 47–49]. A two-step chromatography procedure using Heparin Sepharose and gel filtration is widely used because of its ease of use, high recovery rate, low cost, and ease of scaling up. The procedure that we used to purify rhBSSL from transgenic milk involved two steps: heparin-Sepharose chromatography followed by gel-filtration chromatography. The final purity was of the rhBSSL was 98% with a specific lipase activity virtually identical to that of purified human native BSSL. Importantly, this purification procedure could be scaled up and thus provide a foundation for industrial purification of rhBSSL from transgenic cow milk.

For physiological and biochemical characterization of rhBSSL, it was compared with those of human native BSSL and CHO cells expressed BSSL. Comparison revealed that they have similar physiological and biochemical characteristics. The molecular weight of rhBSSL from transgenic milk appears to be the same as that of CHO-BSSL, and both are smaller than human native BSSL. These differences are likely attribute to different post-translational modifications. It is of note that because of different post-translational modifications, the glycosylation level and pattern of rhBSSL is different compared with that of native BSSL[40]. Even for the native human BSSL, the glycosylation pattern changes during the lactation period in milk of same mother [50]. rhBSSL was confirmed keep stability in absence of bile salt and play the optimal activity under certain concentration of sodium cholate just as human native BSSL[50]. Human Bile salt-stimulated lipase was reported to resistance to acid environment and it could keep stability at pH3.5 for 1 hour[22]. However, in our study rhBSSL and nBSSL are gradually losing their activity between pH3 to pH4. The possible reason is that the buffers prepared had some influence on the formulation of BSSL[51]. rhBSSL also show sensitive to heat and the activity was totally destroy store at 55°C for 30 min. N-terminal sequencing revealed the correct amino acid sequence, indicating that the signal peptide was cleaved correctly. The mature rhBSSL was expressed correctly and secreted into the milk.

In conclusion, we successfully generated rhBSSL transgenic cows with rhBSSL specifically and highly efficiently expressed in the mammary gland and then secreted into milk. In addition, we successfully purified the rhBSSL from the transgenic cow’s milk and achieved a purity of 98% using a two-step chromatographic procedure. The purified rhBSSL maintained its biological activity and exhibited physical and chemical characteristics similar to those of the native protein, e.g., stability at low pH, resistance to inactivation by pancreatic proteases in presence of bile salts, activity at the pH and bile salt concentrations of duodenal content during fat digestion and broad substrate specificity comparable to those of the native human BSSL and CHO cell-derived BSSL. Thus it is suited for oral administration and taken together these characteristics build a solid foundation for further development aiming at improving fat absorption in patients with exocrine pancreatic insufficiency and fat malabsorption for pathological reasons, and improving fat absorption in preterm infants with immature exocrine pancreatic function and thus transient fat malabsorption when fed infant formula which lacks BSSL, or pasteurized human milk in which BSSL has been inactivated by the pasteurization.

## Supporting information

S1 Table Primer sequence for identification. (DOCX)

S2 Table Amino acid compositions of native BSSL and rhBSSL. (DOCX)
