# Supplementary material for: Purification and characterization of recombinant human bile salt-stimulated lipase expressed in milk of transgenic cloned cows
Source: PLoS One. 2017 May 5;12(5):e0176864. doi: 10.1371/journal.pone.0176864 (PMC5419509; doi:10.1371/journal.pone.0176864)
Supplement: S1 Table — (DOCX) [file pone.0176864.s001.docx]

S1 Table. Primer sequence for identification.

| primer | sequence |
| --- | --- |
| BSSL P4-F | 5'- GTCAAGGCGATCTTCAAGTAAAGAC -3' |
| BSSL P4-R | 5'- CGCAATCTCCACCTCCCAGCTTCAA -3' |
| P5-F | 5'-ACAATCCAGGGACATGATACAGA-3' |
| P5-R | 5'-GATGGGACTTTCCGTACAAT-3' |
| P6-F | 5'-CTGTTCAACATTGTACGGAAAGT-3' |
| P6-R | 5'-ATGTCTATACGGGTTATGG-3' |
| P7-F | 5'-CATAACCCGTATAGACATAGGTG-3' |
| P7-R | 5'-GGCCCTCTTAAGTATGTTACAG-3' |
| P8-F | 5'-ACACATTGCCAGAGTAGAGTAGA-3' |
| P8-R | 5'-GCCTTGGTGTCTTAGTATTG-3' |
| P9-F | 5'-ACAGAAGTTATTAGTGCGACCAA-3' |
| P9-R | 5'-GCTACACGGGAAATCTATCTATC-3' |
| P10-F | 5'-GGTCTATTAGAGGCTATGGGTCG-3' |
| P10-R | 5'-GGCCCTCTTAACACTTCC-3' |
