# Supplementary material for: Purification and characterization of recombinant human bile salt-stimulated lipase expressed in milk of transgenic cloned cows
Source: PLoS One. 2017 May 5;12(5):e0176864. doi: 10.1371/journal.pone.0176864 (PMC5419509; doi:10.1371/journal.pone.0176864)
Supplement: S2 Table — (DOCX) [file pone.0176864.s002.docx]

S2 Table. Amino acid compositions of native BSSL and rhBSSL.

| Amino acid | Native BSSL composition (%) | rhBSSL composition (%) |
| --- | --- | --- |
| Asp | 9.21 | 9.63 |
| Glu | 8.68 | 7.2 |
| Ser | 5.61 | 7.55 |
| Gly | 8.36 | 10.24 |
| His | 1.94 | 1.43 |
| Arg | 3.54 | 3.43 |
| Thr | 7.47 | 8.45 |
| Ala | 9.52 | 9.05 |
| Pro | 11.09 | 12.04 |
| Tyr | 3.68 | 3.67 |
| Val | 7.31 | 7.82 |
| Met | 1.26 | 0.4 |
| Cys-cys | 0.13 | 0.04 |
| ILe | 3.83 | 3.5 |
| Leu | 7.86 | 6.51 |
| Phe | 4.05 | 3.43 |
| Trp | - * | - * |
| Lys | 6.46 | 5.61 |

* Trp was destroyed during the analysis
